# Human TRIB2 Oscillates during the Cell Cycle and Promotes Ubiquitination and Degradation of CDC25C

**DOI:** 10.3390/ijms17091378

**Published:** 2016-08-23

**Authors:** Kai Ling Liang, Roberto Paredes, Ruaidhri Carmody, Patrick A. Eyers, Stefan Meyer, Tommie V. McCarthy, Karen Keeshan

**Affiliations:** 1 Paul O’Gorman Leukemia Research Centre, Institute of Cancer Sciences, College of Medical, Veterinary and Life Sciences, University of Glasgow, Glasgow G12 0ZD, UK; 111220352@umail.ucc.ie; 2 School of Biochemistry and Cell Biology, University College Cork, Cork, Ireland; t.mccarthy@ucc.ie; 3 Stem Cell and Leukaemia Proteomics Laboratory, University of Manchester, Manchester M20 3LJ, UK; Roberto.Paredes@manchester.ac.uk (R.P.); stefan.meyer@manchester.ac.uk (S.M.); 4 Institute of Infection, Immunity and Inflammation, College of Medical, Veterinary and Life Sciences, University of Glasgow, Glasgow G12 8TA, UK; ruaidhri.carmody@glasgow.ac.uk; 5 Department of Biochemistry, Institute of Integrative Biology, University of Liverpool, Liverpool L69 7ZB, UK; Patrick.eyers@liverpool.ac.uk; 6 Paediatric and Adolescent Oncology, Royal Manchester Children’s and Christie Hospital, University of Manchester, Manchester M13 9WL, UK

**Keywords:** TRIB2, pseudokinase, CDC25B, CDC25C, dual-specificity phosphatase, ubiquitin proteasome, degradation, cell cycle

## Abstract

Tribbles homolog 2 (TRIB2) is a member of the mammalian Tribbles family of serine/threonine pseudokinases (TRIB1-3). Studies of TRIB2 indicate that many of the molecular interactions between the single *Drosophila* Tribbles (Trbl) protein and interacting partners are evolutionary conserved. In this study, we examined the relationship between TRIB2 and cell division cycle 25 (CDC25) family of dual-specificity protein phosphatases (mammalian homologues of *Drosophila* String), which are key physiological cell cycle regulators. Using co-immunoprecipitation we demonstrate that TRIB2 interacts with CDC25B and CDC25C selectively. Forced overexpression of TRIB2 caused a marked decrease in total CDC25C protein levels. Following inhibition of the proteasome, CDC25C was stabilized in the nuclear compartment. This implicates TRIB2 as a regulator of nuclear CDC25C turnover. In complementary ubiquitination assays, we show that TRIB2-mediated degradation of CDC25C is associated with lysine-48-linked CDC25C polyubiquitination driven by the TRIB2 kinase-like domain. A cell cycle associated role for TRIB2 is further supported by the cell cycle regulated expression of TRIB2 protein levels. Our findings reveal mitotic CDC25C as a new target of TRIB2 that is degraded via the ubiquitin proteasome system. Inappropriate CDC25C regulation could mechanistically underlie TRIB2 mediated regulation of cellular proliferation in neoplastic cells.

## 1. Introduction

The *Tribbles* (*Trbl*) gene was discovered in three independent *Drosophila* genetic screens. Two screens [1,2] were designed to identify mutations that affect gastrulation, the formation of ventral furrow by mesodermal precursor cells during *Drosophila* embryo development. In *Trbl* mutants, the precursor cells exhibit premature mitosis, leading to defective gastrulation. These pioneering studies identified Trbl as an inhibitor of mitosis and implicated Trbl as a direct regulator of fly String function. String is the *Drosophila* orthologue of cell division cycle 25 (CDC25) dual-specificity phosphatases that are required to initiate mitosis and are involved in key cell cycle checkpoint responses. A third screen discovered *Trbl* as one of the genes that affect oogenesis when overexpressed [3]. This study investigated Trbl in *Drosophila* wing and embryonic development, and confirmed that Trbl coordinates mitosis and morphogenesis by promoting proteasomal dependent degradation of String. A recent study demonstrated that Trbl also regulates Twine degradation, a homologue of String, in the *Drosophila* blastoderm during the midblastula transition [4]. Trbl was also found to promote the degradation of Slbo, the *Drosophila* orthologue of the important CCAAT/enhancer binding protein (C/EBP) family of transcription factors, which are critical for transcriptional programmes associated with cell migration during oogenesis [5]. Recently, the proto-oncogene AKT was identified as a third Trbl interacting protein in flies. In this case Trb1 appears to directly inhibit phosphorylation-dependent AKT activation without affecting AKT stability [6]. This is in marked contrast to effects on String and Slbo, where Trbl suppresses function through promotion of proteasome-dependent degradation.

In mammalian systems, three related Tribbles family members (TRIB1-3) are classed as serine/threonine pseudokinases that possess either none, or very low, phosphotransferase capacity [7,8,9]. TRIB proteins contain a pseudokinase domain linked to an ubiquitin E3 ligase targeting motif that has been proposed to interact with the regulatory pseudokinase domain [10]. TRIB proteins are thought to act as pseudokinase scaffold proteins, and are capable of mediating and modulating diverse signalling events that are critical for cellular function and disease pathogenesis [11]. Importantly, the molecular interactions between Trbl and *Drosophila* proteins appear to be evolutionarily conserved in the mammalian system. Like Trbl, TRIB2 mediates the degradation of target proteins including members of C/EBP family. TRIB2-mediated degradation of C/EBPα was found to have an oncogenic role in the development of acute myeloid leukemia (AML) [12,13], and in lung [14] and liver [15,16] models of cancer, whereas TRIB2-mediated degradation of C/EBPβ has been found to suppress adipogenesis in vitro [17]. In addition, TRIB2 blocks adipocyte differentiation by inhibiting phosphorylation-dependent activation of AKT, and this effect was also demonstrated in the *Drosophila* system [17]. Similar to Trbl, TRIB2 has now been shown to regulate cellular proliferation in different cellular contexts [18,19]. However, the molecular mechanism underlying TRIB2 function in cellular proliferation has remained unclear, notwithstanding links to the key cell cycle-regulated CDC25 phosphatases in flies. 

The CDC25 family of proteins are tightly controlled cell cycle master regulators that function as protein phosphatases. They are best characterized as activators of cyclin-dependent kinase (CDK) complexes through dephosphorylation of key inhibitory residues at the N-terminus of the catalytic domain, which in turn promote cell cycle phase progression [20]. The functions of the CDC25 family are highly conserved across species. In Drosophila, String is the orthologue of the CDC25 family [21]. In humans, CDC25 family exists as three related isoforms: CDC25A, CDC25B and CDC25C, all of which are subject to phosphorylation-dependent effects on catalytic activity and stability [22]. CDC25A is thought to promote the G_1_ to S phase transition by activating the CDK2-Cyclin E and CDK2-Cyclin A complexes [23,24] whereas CDC25B/C has been shown to promote G_2_ to M phase transition [25,26]. Nevertheless, under some conditions CDC25A can also regulate G_2_/M phase progression [27], consistent with murine studies that found no apparent cell cycle phenotype in *Cdc25b* single [28], *Cdc25C* single [29] or *Cdc25b/c* double [30] knockout mouse models. The CDC25 family of phosphatases might also have a role in regulation of cell cycle entry/exit (G_0_ to G_1_ transition), as a recently discovered CDC25 inhibitor (NSC 119915), was found to arrest cells in the G_0_/G_1_ and G_2_/M phases of the cell cycle [31]. Finally, regulation of *Cdc25* expression has been implicated in cellular quiescence (G_0_) maintenance and exit in naïve and activated T-cells respectively [32]. However, it is currently unknown whether CDC25 family members can regulate CDK3-Cyclin C complexes, which are required for exit from quiescence [33]. 

In this study, we evaluated human TRIB2-interacting proteins and investigated if the Trbl-mediated degradation of String is likely to be functionally conserved in vertebrate TRIB2 pseudokinase, which have been linked to a variety of cancer-associated signaling pathways [34]. Here, we show that mammalian CDC25B and CDC25C are novel interacting partners of TRIB2. Consistently, the overexpression of TRIB2 promotes polyubiquitination and proteasome-dependent degradation of CDC25C. Our data suggests TRIB2-mediated degradation of CDC25C takes place in the nucleus. In accordance with a potential functional role of TRIB2 in cell cycle regulation, we also find that TRIB2 protein expression is tightly regulated during the cell cycle. 

## 2. Results

### 2.1. TRIB2 Interacts with CDC25B/C But Not CDC25A

**Figure 1:**
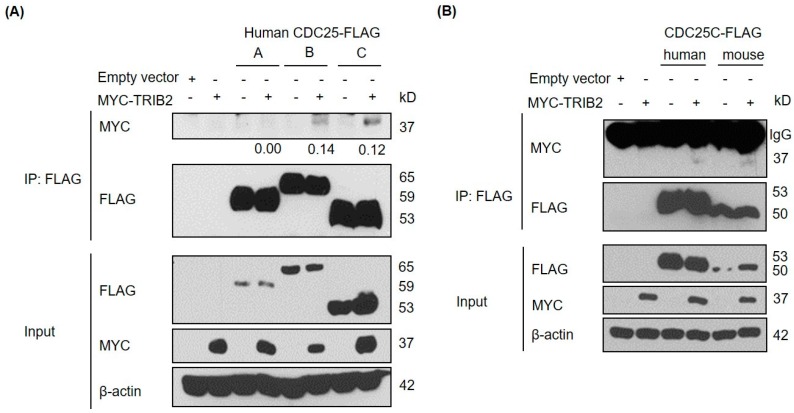
Tribbles homolog 2 (TRIB2) interacts with isoform B and C of cell division cycle 25 (CDC25) family proteins physically. (**A**) Interaction of MYC-tagged TRIB2 with different isoforms of FLAG-tagged CDC25 proteins was examined by co-immunoprecipitation (co-IP) in HeLa cells. IP, immunoprecipitation. Co-immunoprecipitated MYC-tagged TRIB2 signals were quantified by densitometry analyses and normalized to the respective immunoprecipitated FLAG-tagged CDC25 signals. The normalized values are indicated below the sub-panel; (**B**) interaction of MYC-tagged TRIB2 with human and mouse orthologues of FLAG-tagged CDC25C was examined by co-IP.

To examine the interaction of TRIB2 in human cells with all the three isoforms in the CDC25 family, we overexpressed epitope-tagged versions of each protein (FLAG-tagged CDC25A/B/C and MYC-tagged TRIB2) in HeLa cells and performed co-immunoprecitation experiments. Upon immunoprecipitation with anti-FLAG antibody and Western blotting with anti-MYC antibody, we found that TRIB2 co-immunoprecipitated with both human CDC25B and CDC25C but not human CDC25A (Figure 1A) proteins. Hence, TRIB2 interaction with CDC25 family shows some selectivity and, in marked contrast to TRIB3, does not appear to interact with CDC25A [35]. We also demonstrated that TRIB2 binds to both human and mouse CDC25C orthologues (Figure 1B). The weak signal suggests that this is a transient and highly unstable interaction.

### 2.2. TRIB2 Promotes Ubiquitination and Proteasomal Degradation of CDC25C

**Figure 2:**
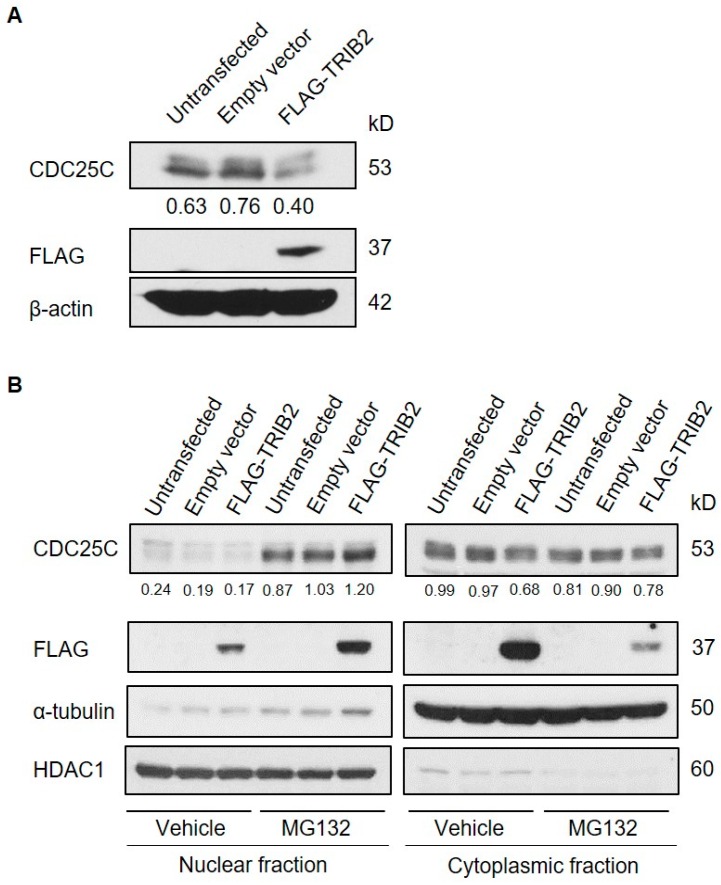
TRIB2 promotes proteasome-dependent degradation of CDC25C in the nucleus. (**A**) Whole cell lysates from FLAG-TRIB2-transfected HeLa cells and controls (untransfected and empty vector-transfected) were analyzed by Western blotting; (**B**) cells were treated with dimethylsulfoxide (DMSO-vehicle) or 10 µM of MG132 for 4 h before subcellular fractionation for Western blotting analysis. α-Tubulin and HDAC1 are cytoplasmic and nuclear markers respectively. CDC25C signals were quantified by densitometry analyses and normalized to the respective loading control signals. The normalized values were indicated below the sub-panel.

To determine if TRIB2 regulates CDC25C through a mechanism related to that described in flies [1,2,3], we examined the effect of TRIB2 overexpression on CDC25C protein expression levels.TRIB2 overexpression has potent leukaemogenic effects, and is associated with proliferation in vitro [18]. Interestingly, overexpression of TRIB2 in HeLa cells led to a decrease in endogenous CDC25C protein expression (Figure 2A), suggesting TRIB2 could regulate CDC25C turnover. In contrast to CDC25A, which primarily resides in the nucleus, CDC25B/C are held inactive in the cytoplasm, and translocate to the nucleus on activation by phosphorylation, in order to promote cell cycle progression [20]. To test the stability of endogenous CDC25C in both nuclear and cytoplasmic compartments in the presence of ectopic TRIB2, we employed the proteasome inhibitor MG132, which prevents degradation of proteasomal substrates. Subcellular fractionation showed that endogenous CDC25C was located primarily in the cytoplasm in untransfected vehicle-cells (Figure 2B). Overexpression of TRIB2 did not affect nuclear translocation of CDC25C proteins in vehicle-treated cells, as expression in the nucleus remained similar when compared to empty vector-transfected cells (Figure 2B). Our data also shows that the treatment of untransfected and empty vector transfected cells with MG132 leads to an accumulation of CDC25C in the nucleus in the absence of a decrease in cytoplasmic CDC25C levels. This strongly argues against the inhibition of CDC25C nuclear to cytoplasmic translocation by MG132 but instead suggests that in the steady state the predominant localization of CDC25C in the cytoplasm may be due to a rapid turnover of nuclear CDC25C. Importantly, in MG132-treated cells, overexpression of TRIB2 led to a marked increase in the level of nuclear CDC25C compared to control untransfected or empty-vector transfected cells (Figure 2B). These data resemble BRCA1 mediated polyubiquitination and proteasomal degradation of CDC25C in response to DNA damage [36]. BRCA1-mediated CDC25C degradation is dependent on nuclear proteasome activity and inhibition of the proteasome in BRCA1 overexpressing cells leads to CDC25C accumulation in the nucleus [36]. Consistently, we found increased CDC25C in the nuclear compartment upon MG132 treatment, suggesting that TRIB2 specifically regulates the turnover of CDC25C in the nucleus. Intriguingly, our data also suggests that TRIB2 stability is also regulated through a similar mechanism (Figure 2B).

**Figure 3:**
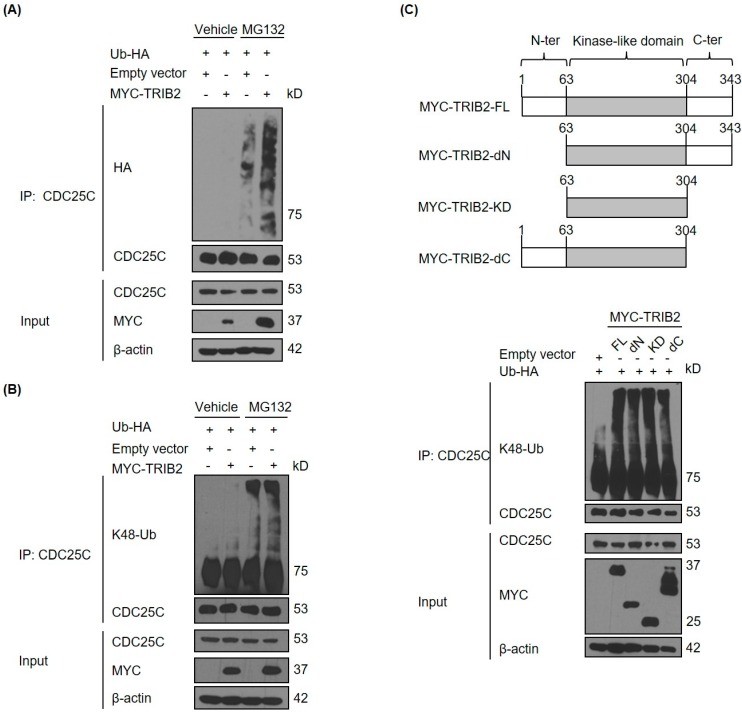
TRIB2 promotes K48-linked polyubiquitination of CDC25C. (**A**) Effect of overexpression of MYC-tagged TRIB2 on ubiquitination of endogenous CDC25C in HeLa cells. Effect of overexpression of (**B**) MYC-tagged TRIB2 wild type and (**C**) different mutants on K48-linked ubiquitination of endogenous CDC25C in HeLa cells. FL, full length; dN, N-terminal deleted; KD, only kinase domain expressed; dC, C-terminal deleted. For (**A**,**C**), all samples were treated with 10 µM of MG132 for 7 h prior cell lysis. Ub-HA, HA-tagged ubiquitin.

TRIB2 has been shown to drive the degradation of proteins via ubiquitination and subsequent recognition of targets through the ubiquitin proteasome system [37]. We therefore sought to determine if TRIB2 has an effect on the ubiquitination of CDC25C in cells. We found that TRIB2 overexpression leads to increased ubiquitination of endogenous CDC25C proteins (Figure 3A). Immunoblotting with an antibody specific for lysine K48-linked polyubiquitin chains demonstrated that TRIB2 promotes the K48-linked polyubiquitination of endogenous CDC25C (Figure 3B). Protein ubiquitination via K48-linked ubiquitin chains is a well-characterised cellular signal for protein elimination through 26S proteasomal degradation [38]. Hence, our results provide the first evidence that mammalian TRIB2 has the ability to promote polyubiquitination of CDC25C, which in turn increases proteasomal dependent degradation of CDC25C phosphatase. TRIB2-mediated degradation of CEBPα requires both an intact kinase-like domain and a C-terminal COP1-binding motif [39]. To determine if specific TRIB2 domains are essential for promotion of endogenous CDC25C ubiquitination, we performed a structure-function analysis of TRIB2 in HeLa cells. As demonstrated with full-length (FL) TRIB2 protein, overexpression of the TRIB2 kinase-like domain (KD) alone was sufficient to drive CDC25C ubiquitination, whereas deletion of either the N or C-terminal regions (dN or dC) had little effect (Figure 3C). Our results confirm that TRIB2 pseudokinase domain is sufficient for TRIB2-mediated ubiquitination and degradation of CDC25C.

### 2.3. TRIB2 Protein Levels Are Regulated in a Cell Cycle Dependent Manner

**Figure 4:**
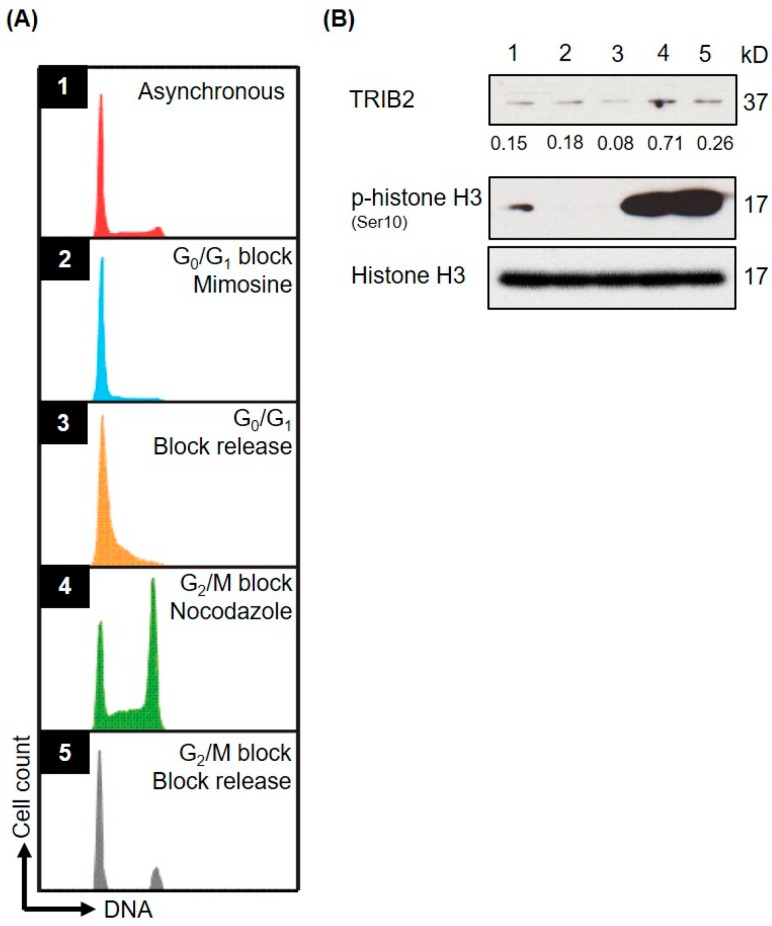
TRIB2 is regulated at the protein level at cell cycle specific phases. Asynchronous (1) SB1690CB cells were G0/G1 (2) arrested by Mimosine followed by release to allow them to progress into S cell cycle phase (3) synchronously and G2/M (4) arrested by Nocodozole followed by release to allow M phase (5) progression. (**A**) Histogram representation of cell cycle analysis by flow cytometry; (**B**) Western blotting analysis with TRIB2 and p-histone H3 signal serves as a mitosis marker. Histone H3 indicated protein loading. TRIB2 signals were quantified by densitometry analyses and normalized to the respective Histone H3 signals. The normalized values were indicated below the sub-panel.

We have previously shown that TRIB2 inhibition either through TRIB2 knockdown or via inhibition of an E2F1-TRIB2 regulatory loop results in perturbation of the cell cycle and cell death [18], and TRIB2 level was shown to be under the control of the β-TRCP ubiquitin E3 ligase in liver cancer cells [16]. Given the new potential role of TRIB2 in the regulation of vertebrate CDC25B/C, we assessed the expression of TRIB2 during cell cycle phases and cell cycle progression. We measured relative TRIB2 protein expression at specific cell cycle phases using a myeloid leukaemia cell line, SB1690CB. G0/G1-S phase border block was achieved by mimosine treatment and G2/M phase border block by nocodazole treatment (Figure 4A). This revealed elevated TRIB2 protein levels in the G2/M phase which co-incided with elevated phosphorylated histone H3 (pSer 10) levels which is a marker of mitosis (Figure 4B).

**Figure 5:**
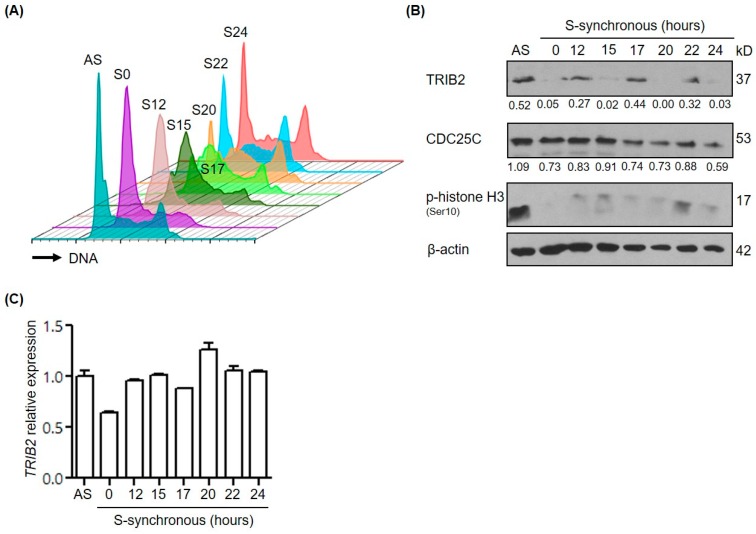
TRIB2 is regulated at the protein level during cell cycle phase progression. Asynchronous (AS) RPMI-8402 cells were arrested by single thymidine block (S0) followed by release (S12–S24) to allow them to progress into different cell cycle phase synchronously. (**A**) Cell cycle analysis by flow cytometry. Histograms of cell cycle profile for all samples were staggered offset; (**B**) Western blotting analysis with p-histone H3 signal serves as a mitosis marker. TRIB2 and CDC25C signals were quantified by densitometry analyses and normalized to the respective β-actin signals. The normalized values were indicated below each sub-panel; (**C**) expression of *TRIB2* mRNA measured by quantitative reverse transcription-polymerase chain reaction (RT-PCR).

To further evaluate the cyclic TRIB2 expression, we synchronized RPMI-8402, a T-cell acute lymphoblastic leukemia (T-ALL) cell line by a single thymidine block, and monitored the level of *TRIB2* mRNA andTRIB2 protein levels as the synchronized cells progressed through the cell cycle following thymidine removal. DNA staining and flow cytometry analysis of samples confirmed the successful synchronization of cells at the G1/S boundary; after removal of thymidine, cells entered and progressed through S phase (S12 and S15) synchronously (Figure 5A). At 20 h after thymidine removal, specific populations of cells were present in G2/M phase (Figure 5A). This was confirmed by detection of increased levels of phosphorylated histone H3 (pSer 10) in cell populations collected at 22 h (S22) (Figure 5B). Remarkably, we observed highly cyclic expression of TRIB2 protein in the absence of significant changes in *TRIB2* mRNA levels (Figure 5B,C). Increased levels of TRIB2 protein were detected in S12, S17 and S22 where cells were synchronized cells in G1/S phase, S phase and M-phase, respectively (Figure 5B). Consistent with our findings, we see reduced total levels of CDC25C at S17 through to S24 where the majority of cells are in S and M-Phase. Hence, TRIB2 protein expression appears to vary cyclically during the cell cycle, which might be relevant to its ability to modulate the stability of CDC25 isoforms.

## 3. Discussion

In this study, we established a novel relationship between mammalian TRIB2 and CDC25 protein family members, and found that TRIB2 interacts physically with CDC25B/C. We showed that TRIB2 promotes CDC25C proteasomal degradation, confirming that this functional relationship has been conserved between Drosopila Trbl [1,2,3] and human TRIB2, which is extremely highly conserved in vertebrates homologues [7]. We found that an increase in CDC25C turnover can be driven by TRIB2-triggered K48-linked polyubiquitination. In addition, our data suggest that TRIB2 degrades a nuclear population of CDC25C, and therefore, TRIB2 may target the key mitotic regulatory fraction of CDC25C. Our analysis reveals cyclic expression of TRIB2 during the cell cycle in-line with an oscillatory cell cycle relationship between TRIB2 and CDC25C. 

Owing to the critical roles of CDC25 family of proteins in cell cycle regulation, the expression and activity of CDC25 proteins is very tightly regulated. CDC25C activity is controlled primarily by two mechanisms at the post-translational level. The first mechanism involves activating or inhibitory phosphorylation of CDC25C, which either leads to CDC25C activation (e.g., through PLK1), or results in binding of CDC25C with 14-3-3 protein and CDC25C sequestration in the cytoplasm [22]. A second mechanism involves inactivation of CDC25C via proteasomal-dependent degradation. Two independently functioning E3 ubiquitin ligases have been so far identified that function independently to regulate CDC25C at distinct cell cycle phase transitions. CDC25C is known to be targeted by APC/C [40,41] for degradation upon mitotic exit, whereas BRCA1 [36] ubiquitinates CDC25C to prevent mitotic entry. We show here for the first time that TRIB2 promotes polyubiquitination and degradation of CDC25C in human cells. It is likely that TRIB2 functions as an adaptor for an as yet unidentified E3 ubiquitin ligase that ubiquitinates CDC25C. TRIB2 has previously been shown to function as an adaptor mediating the degradation of C/EBPα via COP1 E3 ligase in AML [39] or TRIM21 E3 ligase in lung cancer [14], and these are likely candidates that link the TRIB2 pseudokinase domain to ubiquitination.

CDC25C is tightly regulated in the steady state cell cycle as well as in response to stress such as DNA damage that induces G_2_/M checkpoint arrest [22]. Stress-induced checkpoint activation is important to allow cells to repair damaged DNA before resuming cell cycle. Mitogen-activated protein kinase (MAPK) signaling also appears to regulate CDC25C in both steady state cell cycle and checkpoint pathways. During cell cycle at steady state, phosphorylation of CDC25C at Threonine 48 by extracellular signal-regulated kinase (ERK) leads to CDC25C activation and promotion of mitotic entry [42]. However, in response to stress and DNA damage, activated ERK was showed to phosphorylate CDC25C at Serine 216 which in turn promotes CDC25C ubiquitination and proteasomal degradation [43]. In this study, the authors did not show whether the pool of ubiquitinated CDC25C was nuclear or cytoplasmic. C-Jun N-terminal kinase (JNK) was also shown to inactivate CDC25C by phosphorylating CDC25C directly at Serine 168 in order to regulate mitotic entry and G_2_/M DNA damage checkpoint [44]. Lastly, p38 induces CDC25C cytoplasmic sequestration in response to DNA damage thereby inactivating CDC25C indirectly via MK2 kinase [45]. Interestingly, TRIB family members are established to associate with MAPK signalingmodules due to their scaffolding function and capability to interact with MEK1 [46]. Recently, we showed that TRIB2 plays contrasting roles in different subtypes of T-ALL by modulation of MAPK [19]. Further studies are warranted to examine TRIB2/MAPK/CDC25 axis in normal and malignant haematopoiesis, and to evaluate any functional requirements for the related, unstable, TRIB1 [47] and TRIB3 [48] pseudokinases for CDC25-dependent cell cycle and checkpoint signaling pathways.

In conclusion, our study shows for the first time that the function of Trbl-mediated degradation of String is evolutionarily conserved in human TRIB2. Our data confirm TRIB2 selectivity towards different CDC25 isoforms, with TRIB2 interacting with CDC25B and C. Overexpression of TRIB2 promotes K48 linked polyubiquitination and degradation of CDC25C via the nuclear proteasome. The pseudokinase domain of TRIB2 appears to be essential for CDC25C ubiquitination. Our findings provide a novel vertebrate cell cycle link between TRIB2 and CDC25C, and suggest regulated TRIB2 functions during the cell cycle that are likely to be important in cancer.

## 4. Materials and Methods

### 4.1. Cell Lines

RPMI-8402 cell line was obtained from Leibniz Institute DSMZ (German Collection of Microorganisms and Cell Cultures, Braunschweig, Germany), and HeLa cell line obtained in-house. Both cell lines were cultured in RPMI 1640 medium (Gibco, Paisley, UK) supplemented with 2 mM l-glutamine (Gibco), 10% fetal bovine serum (Sigma Aldrich, Dorset, UK) and 100 units·mL^−1^/100 μg·mL^−1^ Penicillin/Streptomycin (Gibco). SB1690CB cell line was established and cultured as described previously [49]. All cultures were maintained in a tissue incubator at 37 °C under 5% of carbon dioxide and were free from mycoplasma contamination.

### 4.2. Plasmids and Transfection

Plasmids that encode Ub, TRIB2 wild type and mutants have been described [13,39]. CDC25A/B inserts from pCMV6-CDC25A/B-MYC-FLAG (OriGene, Rockville, MD, USA: RC200496 and RC207409) were sub-cloned into MluI/AsiSI digested pCMV6-AC-FLAG empty vector (OriGene). Human CDC25C insert was PCR-amplified from pcDNA3-HisA-CDC25C [50] (Addgene plasmid #10964) and was ligated into MluI/AsiSI digested pCMV6-AC-FLAG. Cells were transfected with plasmids indicated in the related figures using X-tremeGENE™ HP DNA transfection reagent (Roche, Dorset, UK).

### 4.3. Antibodies and Western Blotting

The following antibodies were used for Western blotting: Anti-K48-Ub (Apu2 clone; Millipore, Watford, UK), anti-phospho-Histone H3 (D2C8 clone; Cell Signaling Technology, Leiden, The Netherlands and #06-570; Millipore), anti-Histone H3 (#9715; Cell Signaling Technology) anti-α-tubulin (B-5-1-2 clone; Sigma Aldrich), anti-β-actin (AC-15 clone; Sigma Aldrich), anti-FLAG (M2 clone; Sigma Aldrich), anti-HA (HA-7 clone; Sigma Aldrich), anti-CDC25C (C-20 clone; Santa Cruz Biotechnology, Heidelberg, Germany), anti-HDAC1 (H-51 clone; Santa Cruz Biotechnology), anti-MYC (9E10 clone; Santa Cruz Biotechnology) and anti-TRIB2 (B-06 clone; Santa Cruz Biotechnology). Unless indicated otherwise, Western blotting was performed as described previously [19]. Densitometry analyses were performed using Image J (NIH, Bethesda, MD, USA) [51].

### 4.4. Co-Immunoprecipitation

Cells were lysed in protease inhibitors-supplemented Tris lysis buffer (50 mM Tris buffer (pH 7.4), 150 mM NaCl, 0.5% IGEPAL^®^ CA-630, 5% glycerol and 1 mM EDTA) after 24 h of transfection. Prior to immunoprecipitation, protein lysates were pre-cleared by incubation with Protein G Agarose beads (Millipore, Cork, Ireland) at 4 °C for 30 min. For immunoprecipitation, pre-cleared lysates were incubated with the indicated antibody and Protein G Agarose beads at 4 °C for overnight. All incubations were done on a rotary tube mixer. Immunoprecipitated proteins were eluted from washed beads by boiling at 95 °C for 5 min in Laemmli buffer and analyzed by Western blotting.

### 4.5. Subcellular Fractionation

Following transfection, cells were treated with DMSO (Sigma Aldrich) or 10 μM MG132 (Sigma Aldrich) for 4 h at 37 °C before subcellular fractionated by Active Motif Nuclear Extract Kit (La Hulpe, Belgium). Nuclear and cytoplasmic lysates were analyzed by Western blotting.

### 4.6. Ubiquitination Assay

Following transfection, cells were treated with dimethylsulfoxide (DMSO) or 10 μM MG132 for 7 h at 37 °C followed by 10 mM *N*-ethylmaleimide (Sigma Aldrich) for 30 s at room temperature. Cells were then lysed in 1% (wt/vol) sodium dodecyl sulphate (Sigma Aldrich) solution by boiling at 95 °C for 5 min. The crude cell lysate was sonicated prior centrifugation to derive cleared protein lysate. Protein lysate was diluted in protease inhibitors-supplemented Tris lysis buffer (50 mM Tris buffer (pH 7.4), 150 mM NaCl, 0.5% IGEPAL^®^ CA-630, 5% glycerol and 1 mM EDTA). Pre-clearing, immunoprecipitation and elution of immunoprecipitated proteins were performed, as described in Section 4.4. Protein ubiquitination was analyzed by Western blotting.

### 4.7. Cell Cycle Synchronization

SB1690CB cells were arrested in late G_1_ phase with 0.2 mM mimosine (Sigma Aldrich) for 24 h or in G_2_/M phase with 200 ng/mL of nocodazole (Sigma Aldrich) for 18 h. G_1_ and G_2_/M phase-arrested cells were released from block by replacing medium and culturing them further at 37 °C for 7 h. Samples were collected at different points of the cell cycle for flow cytometry and Western blotting analyses. For Western blotting, cells were lysed in protease inhibitors-supplemented CHAPS/Bicine buffer (20 mM bicine, 0.6% CHAPS, 2 mM MgCl_2_, 1 μM ZnCl_2_, 420 mM NaCl and 500 U/mL Pierce Universal Nuclease).

RPMI-8402 cells were sub-cultured a day before they were treated with 0.75 mM thymidine (Sigma Aldrich) and cultured further at 37 °C for 21 h. Synchronous cells (S0) were then released from thymidine block by washing twice in phosphate buffered saline (PBS) before resuspended in fresh complete medium for further culture. Samples were collected at different time points (S12, S15, S17, S20, S22 and S24) for flow cytometry, Western blotting and quantitative reverse transcription-polymerase chain reaction (RT-PCR) analyses. For Western blotting, cells were lysed directly in Laemmli buffer.

### 4.8. Cell Cycle Analysis

Cells were fixed in 70% ice-cold ethanol and stored at −20 °C. To analyze cell cycle, cells were washed twice in PBS before they were resuspended in BD Pharmingen™ Propidium Iodide/RNase Staining buffer (BD Biosciences, Oxford, UK). Flow cytometry was performed using BD FACSCanto II system (BD Biosciences). Aggregates were excluded by gating of forward scatter height versus area signals.

### 4.9. Quantitative RT-PCR

RNA was extracted from cells using RNeasy^®^ Mini Kit (Qiagen, Manchester, UK) and converted to cDNA using High Capacity cDNA Reverse Transcription Kit (Applied Biosystems, Paisley, UK). *TRIB2* and *β2M* mRNA expressions were measured using Fast SYBR^®^ Green Master Mix (Applied Biosystems). Primer sequences have been described [19]. Each target was measured in triplicate reactions. *TRIB2* expression was normalized to *β2M* and was calculated using the 2^−ΔΔ*C*t^ method.

## Abbreviations

AML	Acute myeloid leukemia
CDC25	Cell division cycle 25
C/EBP	CCAAT/enhancer binding protein
DMSO	Dimethylsulfoxide
ERK	Extracellular signal-regulated kinase
JNK	c-Jun N-terminal kinase
KD	kinase-like domain
MAPK	Mitogen-activated protein kinase
PBS	Phosphate buffered saline
RT-PCR	Reverse transcription-polymerase chain reaction
T-ALL	T-cell acute lymphoblastic leukemia
Trbl	*Drosophila* Tribbles
TRIB2	Tribbles homolog 2
Ub-HA	Ub-tagged ubiquitin
